# Basophil Activation Test identifies the patients with Chronic Spontaneous Urticaria suffering the most active disease

**DOI:** 10.1002/iid3.125

**Published:** 2016-10-04

**Authors:** Laia Curto‐Barredo, Jose Yelamos, Ramon Gimeno, Sergi Mojal, Ramon M. Pujol, Ana Giménez‐Arnau

**Affiliations:** ^1^ Department of DermatologyInstitut Hospital del Mar d'Investigacions Mèdiques (IMIM), Universitat AutònomaBarcelonaSpain; ^2^ Department of ImmunologyInstitut Hospital del Mar d'Investigacions Mèdiques (IMIM)BarcelonaSpain; ^3^ Department of StatisticsInstitut Hospital del Mar d'Investigacions Mèdiques (IMIM)BarcelonaSpain

**Keywords:** autologous serum skin test, basophil activation test, chronic spontaneous urticaria

## Abstract

**Introduction:**

The basophil activation test showing CD63 up regulation could be a specific and sensitive in vitro complementary text to the in vivo autologous serum skin test for the activity assessment of the patients suffering autoimmune chronic spontaneous urticaria. The aim of this study is to define the basophil activation test as a useful tool in clinical practice in order to identify those patients with more active disease.

**Methods:**

We screened 139 patients (96 women) diagnosed of chronic spontaneous urticaria using simultaneously autologous serum skin test and basophil activation test and their relationship with disease activity.

**Results:**

Positive autologous serum skin test was found in 56.8%; from them, 31.6% were basophil activation test positive. Negative autologous serum skin test result was found in the 43.2% of the sample that showed negative CD63 expression results in all cases, except one. Patients with positive autologous serum skin test and positive CD63 by basophil activation test showed significant higher Urticaria Activity Score of 7 days (*P* = 0.004) and of 3 weeks (*P* = 0.001) than patients with positive autologous serum skin test and negative CD63 (mean ± standard deviation [SD] 26.57 ± 10.56 versus 18.40 ± 12.05 for the Urticaria Activity Score of 7 days and 56.47 ± 23.78 versus 39.88 ± 25.44 for the Urticaria Activity Score of 3 weeks).

**Conclusions:**

The CD63 expression on basophils appears as a reliable in vitro marker, useful in clinical practice in combination with autologous serum skin test to define chronic spontaneous urticaria patients with the highest urticaria activity that impairs a normal life.

## Introduction

Chronic spontaneous urticaria (CSU) is defined as the appearance of evanescent wheals; angioedema or both that persist for longer than 6 weeks. It is the most common subtype of chronic urticaria, having a significant impact in quality of life 1. The clinical and serologic criteria defining autoimmune CSU are still a matter of research 2. The in vivo autologous serum skin test (ASST) is the diagnostic tool for evaluation of serum autoreactivity mostly due to autoantibodies against the high‐affinity immunoglobulin E (IgE)‐receptor (FcϵR1α) or IgE itself in patients with CSU 3. The basophil histamine release assay (BHRA), when is positive is defined as one of the criteria of autoimmune CSU and its assessment is recommended 4. BHRA is a safe and reliable diagnostic tool, but is usually not routinely available in our daily clinical practice.

In recent years, the quantification of basophil activation by flow cytometry (basophil activation test, BAT) has been proposed as a proven useful tool for the assessment of responses to allergens mediated by IgE. In such test, the patient serum is coincubated with donor basophils and the activation is measured. Most BAT studies have used CD63 or CD203c as good basophil activation markers after antigen‐specific stimulation 3. The CD63 molecule is a tetraspan granule protein that is not expressed on resting basophils but is up regulated after its activation. BAT with CD63 up regulation has been established as a specific and sensitive in vitro alternative to ASST to identify functional autoantibodies (specific immunoglobulin G (IgG) binding IgE or its high affinity receptor) in CSU 5, 6, 7. Some heterogeneous results have been published in relation to methodological differences using different basophil donors. BAT offers some advantages compared with the ASST to be implemented in clinical practice providing quantifiable results suitable to monitor the course of the disease and its treatment 8. The possibility that BAT may replace ASST in a near future as a diagnostic standard procedure to identify autoreactive serum factors in CSU has been postulated 9.

Our objective was to show if the use of BAT routinely, in combination with ASST, in daily clinical practice of management of patients with CSU could help us confirm those patients with more active disease.

## Materials and Methods

### Patients and urticaria assessment

We aimed to assess the diagnostic practical usefulness of the simultaneous evaluation of ASST and BAT in 139 patients with CSU (96 women and 43 men, mean age ± standard deviation [SD]): 45.5 ± 14.7) and their relationship with activity of the disease, measured using the Urticaria Activity Score 10 from the day before (UAS; 0–6), the 7 days before (UAS7; 0–42) and the 3 weeks before (UAS3w; 0–126) the baseline clinical assessment. The UAS7 (0–42) is a validated patient report outcome including two parameters number of wheals (0–21) and intensity of itch (0–21). The UAS from the 3 weeks before helps to assess such patients with active CSU partially controlled that maybe do not show active symptoms immediately before the medical consultation but CSU is still active.

The diagnosis of urticaria was based on clinical history data and physical examination. Those patients with urticaria vasculitis or other urticarial syndromes different from urticaria were excluded. All patients aged more than 16 years with a diagnosis of active CSU from at least the previous 6 months. All patients were studied prospectively according with the complete protocol for CSU, following the European guidelines 11. Diagnostic interventions were informed and approved by the patients. At the baseline assessment the patients were free of any active treatment. A blanching period of at least 15 days for antihistamines, 3 weeks for any immunosuppressive, and 30 days for anti‐IgE treatment was guaranteed. Patients diagnosed of CSU showing an active disease along the 4 weeks before were included. Study 2013/5363/I approved by the ethic committee, CEIC‐Parc de Salut Mar, IMIM‐Hospital del Mar.

### Combined use of the ASST and the BAT

Positive reaction for ASST was defined when the diameter of serum‐induced wheal was >1.5 mm compared to saline‐induced response at 30 min 12. Total IgE levels were measured by the ImmunoCAP system and the anti‐thyroglobulin and thyroperoxydase antibodies were detected by radioimmunoassay as part of the routine study protocol of all CSU patients.

BAT was performed, measuring expression of the CD63 surface marker on basophils from whole blood by flow cytometry using a commercial kit (Flow2CAST, Bühlmann laboratories) according to the manufacturer's instructions. The basophils used for this test were obtained from different healthy donors.

Briefly, within 2 h after venipuncture, 50 μl of EDTA‐blood were mixed with: (i) 50 μl of patient serum or 50 μl of healthy serum (negative control) or 50 μl of a solution containing anti‐FcϵRI monoclonal antibody (positive control‐1 provided by the kit), or 50 μl of a solution containing fMLP (positive control‐2 provided by the kit); (ii) 100 μl of stimulation buffer containing calcium, heparine, and IL‐3 13 (provided by the kit); (iii) and 20 μl of a mix of FITC‐conjugated anti‐CD63 and PE‐conjugated anti‐CCR3 monoclonal antibodies (provide by the kit). After incubation for 15 min at 37°C, samples were lysed and washed with reagents provided by the kit and cells were adquired in a FACS Canto cytometer (Beckton–Dickinson, San Jose, CA). Basophils were identified as CCR3+‐cells with low site scatter SSC. At least 500 basophils were acquired and analysed for CD63 expression. Two conditions were required to consider a BAT positive: (i) the percentage of CD63+ basophils >5%, and (ii) the stimulation index (SI = % CD63+ induced by the serum patient divided by % CD63+ induced by the negative control) must be equal or higher than 2.

This selection led us to apply this technology in the routine clinical practice beyond the research field.

### Statistic assessment

The relationship between the obtained results and the severity of the disease was evaluated statistically by using an unpaired two‐tailed Mann–Whitney test. All the descriptive and inferential analyses were done by means of the SPSS program (version 15). P values < 0.05 were considered significant.

## Results

The clinical features, severity scores and main results of ASST and BAT are summarized in Table 1. From the total sample, the mean ± SD of serum IgE was 145.38 ± 150 (1.3–909) kU/L, slightly above of the normal values (0–100 kU/L) as it was expected. This finding was observed for the first time during the omalizumab trials and its clinical relevance, especially for free‐IgE levels is still a matter of study but according with the role of IgE and FcϵR1α in CSU 14. Twenty‐two percent of the patients who were tested for thyroid function had clinical or subclinical thyroid disease. This association between autoimmune thyroid disease and CSU is well known and is one of the clinical associations that contributed to build the autoimmune hypothesis 15, 16.

**Table 1:** Demographic and characteristics of CSU activity

Total sample size, *n* = 139^1^	
Female/Male, *n* (%)	96 (69.1)/43 (30.9)
IgE (range in kU/L), mean ± SD	145.38 ± 150 (1.3–909)
Age (y), mean ± SD	45.5 ± 14.7
Thyroid disease, *n* (%)	23/101 (22.8)
Autoimmunity, *n* (%)	19/23 (82.6)
Subclinic disease, *n* (%)	11/19 (57.9)
Hypothyroidism, *n* (%)	7/19 (36.8)
Hyperthyroidism, *n* (%)	1/19 (5.3)
**Groups by ASST/CD63 results**	
**ASST +/ CD63 + subgroup (*n* = 25), mean ± SD (range)**	
UAS 4.26 ± 1.84 (1–7)	
UAS7 26.57 ± 10.56 (7–42)	
UAS3w 61.87 ± 25.62 (21–120)	
**ASST +/ CD63‐ subgroup (*n* = 54), mean ± SD**	
UAS 2.92 ± 1.75 (0–6)	
UAS7 18.40 ± 12.05 (0–49)	
UAS3w 39.88 ± 25.44 (10–120)	
**ASST‐/CD63‐ subgroup (*n* = 59), mean ± SD**	
UAS 2.93 ± 2.22 (0–12)	
UAS7 17.28 ± 12.61 (0–48)	
UAS3w 38.39 ± 28.59 (15–120)	

ASST, autologous serum skin test; CD63, basophil activation test; SD, standard deviation; UAS, Urticaria Activity Score/the day before; UAS7, Urticaria Activity Score the week before; UAS3w, Urticaria Activity Score 3 weeks before; y, years.

From the 139 patients with ASST performed, the UAS was not available in nine patients. And one patient showed negative ASST and positive CD63.

CSU Categorical Health States attending the UAS7 results: no urticaria (UAS 7 = 0), well controlled activity (UAS7: 1–6), mild activity (UAS7: 7–16), moderate activity (UAS7:17–27), severe activity (UAS7: 28–42) 20.

^1^ One patient showed positive CD63 and negative ASST.

Positive skin reactivity (positive ASST) was found in 56.8% of the sample in agreement with previous published data of positive ASST frequency ranges 17. The high percentage of positivity in ASST can be explained by the fact, that our Urticaria Unit, assesses CSU patients habitually very active and commonly refractory to conventional treatments. From that positivity in ASST patients, 31.6% showed a positive BAT as determined by expression of CD63 and 68.4% were negative. ASST negative result was found in the 43.2% of the sample that showed negative CD63 expression results in all cases, except one case. In our hands, the ASST previously performed in 22 healthy controls was always negative.

**Figure 1:**
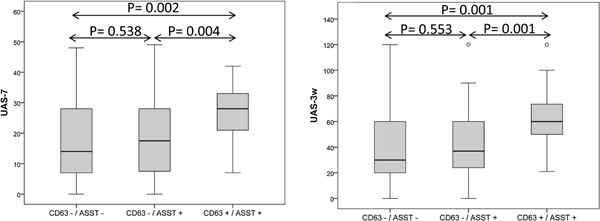
Box‐plots representing the severity of the disease, by positive and negative ASST and CD63 groups. Activity of the Urticaria was recorded at three different times: the day before (UAS), week before (UAS7), and 3 weeks before (UAS3w). This Figure shows UAS7 and UAS3w assessment by groups. Black horizontal line segment inside the box represents the median. Bottom and top box lines represent the 25th and the 75th percentiles, respectively.

Patients with positive ASST and whose sera induced CD63 expression on heterologous basophils showed significantly higher scores in UAS (mean ± SD 4.26 ± 1.84, *P* = 0.003), UAS7 (26.57 ± 10.56, *P* = 0.002), and UAS3w (61.87 ± 25.62, *P* = 0.001) than patients with negative ASST and CD63 results. Patients with positive ASST and positive CD63 expression by BAT showed higher UAS and UAS7 (*P* = 0.004 in both cases) and UAS3w (*P* = 0.001) than positive ASST and negative CD63 expression (ASST+/CD63‐ group: UAS 2.92 ± 1.75, UAS7 18.40 ± 12.05, UAS3w 39.88 ± 25.44) (Fig. 1). No significant differences on the UAS, UAS7, or UAS3w were observed just assessing positive ASST neither versus negative ASST patients nor versus positive ASST and negative patients once the expression of CD63 by BAT was negative. All the patients with positive BAT, except one were positive for ASST, resulting in a high specificity of 98.3%. However, only 31.6% of the serum of all the patients with positive ASST induced CD63 expression (sensitivity of 31.6%). In addition, all healthy donors sera (*n* = 9) showed a negative CD63 expression by BAT.

## Discussion

The current results in this experimental approach show that a 68.4% of the in vivo autoreactive serum samples did not activated the basophils from healthy donors and that the serum of 31.6% of all the patients with positive ASST induced a CD63 expression on heterologous basophils. This percentage is low compared with other previous studies that showed an up‐regulation of CD63 expression in 70% of patients with positive ASST 6. But this refered study, report a 45% of negative ASST patients with positive CD63 expression, this fact imply decreased rate of specificity. The specificity in our study is of 98.3% comparable with the 90.5% described previously 18. However, very low 31.6% sensitivity is observed as compared with the 95.5% found in other studies that include less number of samples and just two selected donors of basophils 18. These differences can be explained partially by the basophil donor variability (healthy subjects) in our study. In our study, there was not a selection of a specific donor example a special population such as atopic patients and we used different healthy donors. Interestingly, patients with negative ASST showed a negative BAT independently of the activity of CSU. So, our finding indicates that the CD63 expression by BAT provides no false‐positive results and was limited to those patients with serum autoreactivity, demonstrating high specificity. Significantly, the CSU patients with positive ASST whose serum induced expression of CD63 showed the most active disease (higher UAS scores) being suitable to use high dose of antihistamines joint with third line treatment. The percentage of patients ASST+/CD63+ that required third line of treatment according with the European guidelines (Cyclosporine A or Omalizumab) was in our study the 32% compared with the 16.66% ASST+/CD63‐. When ASST‐/CD63‐ a 30.50% of patients required also third line of treatment suggesting other mechanisms involved in the development of the wheals than those involved in autoimmunity.

The basophil activation test through CD63 expression might be a reliable and safe test helping to identify the most active CSU patients showing positive autoreactive serum. The CD203c molecule has been proposed as an alternative in chronic urticaria patients with severe disease that could be used also as a severity marker 19; however, this marker led to variable activation patterns that are better controlled by CD63 when healthy basophil donors are used. A significant correlation between the basophil histamine release and the CD63 assays was observed using basophils of two selected no atopic and atopic donors, and the ASST showed a strong correlation with the histamine release only when the basophils were from an atopic donor 20.

The BAT test offers some advantages over ASST that must be taken into consideration in clinical practice. Some of these advantages would be: it is a valid technique to identify patients expressing anti‐IgE antibodies and anti IgE receptor, its good correlation with CSU activity would allow disease treatment monitoring, it is a technique less invasive and applicable without problem at any age.

Despite the variability in basophil donors, our good coincidence between tests gains special relevance in the context of the clinical routine in order to implement the CD63 without the need of a selection of high sensitive basophil donor to favour the CD63 expression and the profitability of the technique, as most studies do 6, 20; thus, we get close an indicator for routine diagnostics. These results allow us to recommend the implementation of the BAT technique routinely with reliable results (assuming all the possible limitations). According to our results, the assessment of the CD63 expression by BAT may be considered as an in vitro biological marker of disease activity.

BAT technique helped us to confirm in our daily practice the identification of the most active patients suffering CSU according with UAS7. Although, the UAS7 is a valid instrument it is a patient report outcome it depends on the opinion and the will of the patient. A battery of useful biomarkers in clinical practice would be necessary and is desirable in CSU. In the search for clinical features and laboratory findings that would help us to identify and confirm the more active patients suffering of CSU, BAT can be considered a useful tool.

### Conflict of Interest

None declared.
